# Engineered AAA+ proteases reveal principles of proteolysis at the mitochondrial inner membrane

**DOI:** 10.1038/ncomms13301

**Published:** 2016-10-27

**Authors:** Hui Shi, Anthony J. Rampello, Steven E. Glynn

**Affiliations:** 1 Department of Biochemistry and Cell Biology, Stony Brook University, Stony Brook, New York 11794-5215, USA

## Abstract

The human YME1L protease is a membrane-anchored AAA+ enzyme that controls proteostasis at the inner membrane and intermembrane space of mitochondria. Understanding how YME1L recognizes substrates and catalyses ATP-dependent degradation has been hampered by the presence of an insoluble transmembrane anchor that drives hexamerization of the catalytic domains to form the ATPase active sites. Here, we overcome this limitation by replacing the transmembrane domain with a soluble hexameric coiled coil to produce active YME1L hexamers that can be studied *in vitro*. We use these engineered proteases to reveal principles of substrate processing by YME1L. Degradation by YME1L requires substrates to present an accessible signal sequence and is not initiated simply by substrate unfolding. The protease is also capable of processively unfolding substrate proteins with substantial thermodynamic stabilities. Lastly, we show that YME1L discriminates between degradation signals by amino acid composition, implying the use of sequence-specific signals in mitochondrial proteostasis.

## Abstract (web summary)

Human YME1L is a membrane-anchored AAA+ protease that maintains proteostasis in the mitochondrial inner membrane and intermembrane space. Here the authors probe the substrate-binding and degradation activities of YME1L and suggest the existence of sequence-specific degradation signals in mitochondrial proteostasis.

Mitochondria play crucial roles in regulating eukaryotic energy production, calcium signalling and programmed cell death^1^,^2^. These activities are carried out by a composite mitochondrial proteome encoded by both the nuclear and mitochondrial genomes^3^,^4^,^5^,^6^. Mitochondrial proteins face constant oxidative threat from reactive oxygen species, by-products of the respiratory processes that occur at the mitochondrial inner membrane^7^. Preserving mitochondrial function in a hostile environment requires robust systems of protein quality control to maintain proper protein levels, remove damaged respiratory components and balance mitochondrial fusion and fission^8^,^9^. Indeed, misregulation of the mitochondrial proteome has been implicated in the development of severe human diseases such a diabetes, cancer and neurodegenerative disorders^1^,^10^,^11^.

In all mitochondrial compartments, proteostasis is accomplished by the AAA+ family of proteases that capture energy from ATP to catalyse protein degradation. Multiple subunits assemble to form a peptidase chamber bearing narrow entrances that require substrate proteins to be unfolded before entry^12^,^13^. Substrate unfolding is catalysed by a ring of AAA+ ATPase domains that stack upon the peptidase chamber and select which proteins within a crowded cellular environment are delivered to the peptidase for degradation (Fig. 1a). Upon binding to a substrate, ATP-driven conformational fluctuations in the AAA+ ring deliver an unfolding force to the substrate followed by translocation of the unfolded polypeptide into the peptidase chamber^14^,^15^,^16^. Substrate interaction is commonly achieved by the recognition of degradation signals (degrons) present within the substrate^13^. Degrons can take the form of specific amino acid sequences found at either terminus or internally within the polypeptide (for example, the bacterial ssrA tag^13^), defined structural architectures (for example, the tetrameric arrangement of the Mu transposase complex^17^,^18^) or covalently linked accessory proteins (for example, polyubiquitin recognition by the 26S proteasome^19^). Understanding protein degradation by a AAA+ protease requires probing both the affinity of the protease for the degron to achieve satisfactory substrate binding and the ability of the protease to apply sufficient force to initiate unfolding.

Two homologous AAA+ proteases are anchored to the mitochondrial inner membrane where they regulate the composition and quality of the mitochondrial proteome in the intermembrane space (IMS), inner membrane and matrix (Fig. 1a). The i-AAA protease (YME1L in humans) assembles into hexamers of six identical subunits, each containing a short N-terminal domain, a single transmembrane span, a AAA+ ATPase domain, and a zinc metalloprotease domain belonging to the M41 peptidase family (Fig. 1b)^20^,^21^. Insertion of the insoluble transmembrane span into the inner membrane projects the soluble ATPase and protease domains into the aqueous IMS where they are positioned to engage both soluble proteins and solvent exposed regions of integral inner membrane subunits^22^. Alternatively, the m-AAA protease in humans can assemble as either a homo- or heterohexamer with the catalytic domains projected into the mitochondrial matrix. The opposing orientations of the two proteases allow the IMS, matrix and both faces of the inner membrane to be scrutinized for the emergence of proteins requiring removal (Fig. 1a)^22^,^23^.

Known substrates of YME1L and its homologues include the inner membrane-anchored regulator of mitochondrial dynamics, OPA1 (ref. 24); soluble intermembrane space proteins (for example, the small translocases Tim9 and Tim10 (ref. 25)) and integral membrane components of the electron transport chain (for example, Cox2 (ref. 26)). How this diverse group of substrates are recognized and engaged by YME1L is unclear. Given the abundance of degrons found across substrates of other AAA+ family members, it is likely that proteins degraded by YME1L will contain specific sequences that are recognized by the protease. However, no such motifs have yet been identified. Interestingly, *in vivo* degradation of chimeric fusions of an integral inner membrane protein with destabilized mouse dihydrofolate reductase (mDHFR) suggested that the yeast protease (Yme1p) may recognize simple unfolded structures or sequences that become accessible upon substrate unfolding, with a minimum length of 10–20 residues required to project from the inner membrane face^23^,^27^.

An important question is whether mitochondrial AAA+ proteases are capable of delivering a significant pulling force. The unfolding force of different AAA+ proteases is variable and has been correlated with both the rate of ATP hydrolysis and the gripping of the central pore loops to the substrate polypeptide^28^,^29^,^30^. Several lines of evidence suggest that the mitochondrial proteases may not unfold stable proteins. Firstly, the related bacterial membrane-anchored protease, FtsH, is incapable of unfolding highly stable proteins but can degrade low stability or destabilized proteins, possibly as a mechanism for achieving preferential degradation of damaged substrates^31^. Secondly, Yme1p has been shown to degrade both nonassembled mitochondrial proteins Cox2 and Phb1 (refs 26, 32). Thirdly, chimeric membrane-anchored proteins are degraded by Yme1p only when bearing substitutions that favor destabilization of the substrate's intermembrane space domains or at high temperature^23^,^27^. Alternatively, this result could be explained by the appearance of accessible internal degrons after substrate destabilization. Degradation of any membrane-spanning substrate necessarily requires the generation of a significant pulling force to dislocate the hydrophobic polypeptide from the membrane.

Elucidation of the mechanisms that the mitochondrial AAA+ proteases use to recognize and process protein substrates requires precise measurements in the absence of competing enzymes and substrates. Our current understanding of these enzymes is largely built on a number of elegant *in vivo* studies in yeast and assumptions taken from *in vitro* studies of related family members. Attempts to produce soluble active mitochondrial AAA+ proteases by removal of the transmembrane span have been unsuccessful, as these variants do not form hexamers, hydrolyse ATP, or carry out ATP-dependent proteolysis^27^. Here, we report the development of a system for assembling membrane-anchored AAA+ proteases in a soluble, active, hexameric form. This has enabled the first analysis of the substrate binding and unfolding capacities of human YME1L *in vitro*. These experiments reveal that simple unfolding of a protein substrate is not sufficient to initiate degradation by YME1L but that degradation can be stimulated by the addition of a degron sequence to a terminus. We show that YME1L degrades substrates processively from the degron terminus and can generate a significant power stroke that is capable of unfolding stable proteins. In conclusion, we show that YME1L discriminates between degrons by sequence identity, implying that mitochondrial substrates are recognized through specific motifs.

## Results

### Engineering an active YME1L protease *in vitro*

**Figure 1:**
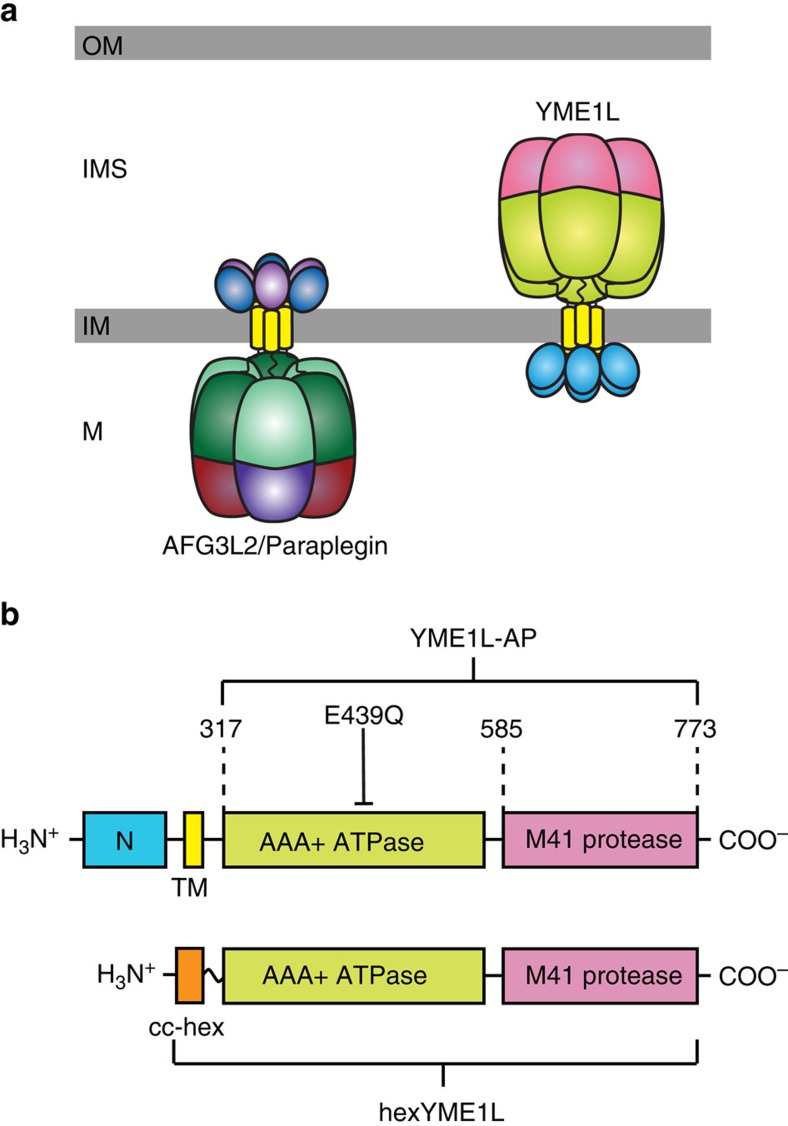
AAA+ proteases at the mitochondrial inner membrane. (**a**) YME1L and AFG3L2/Paraplegin are two homologous human AAA+ proteases anchored to the inner membrane of mitochondria where they degrade protein substrates in both soluble compartments and the inner membrane (OM=outer membrane; IMS=intermembrane space; IM=inner membrane; M=matrix). (**b**) Each YME1L subunit contains an N-terminal domain (blue), transmembrane span (yellow), AAA+ ATPase domain (green) and M41 zinc metalloprotease domain (pink). The YME1L-AP construct contains the AAA+ ATPase domain and M41 protease domain (residues 317–773). In hexYME1L, the N-terminal domain and transmembrane span are replaced by the 30-residue cc-hex coiled coil sequence (orange). The E439Q substitution blocks ATP hydrolysis activity.

**Figure 2:**
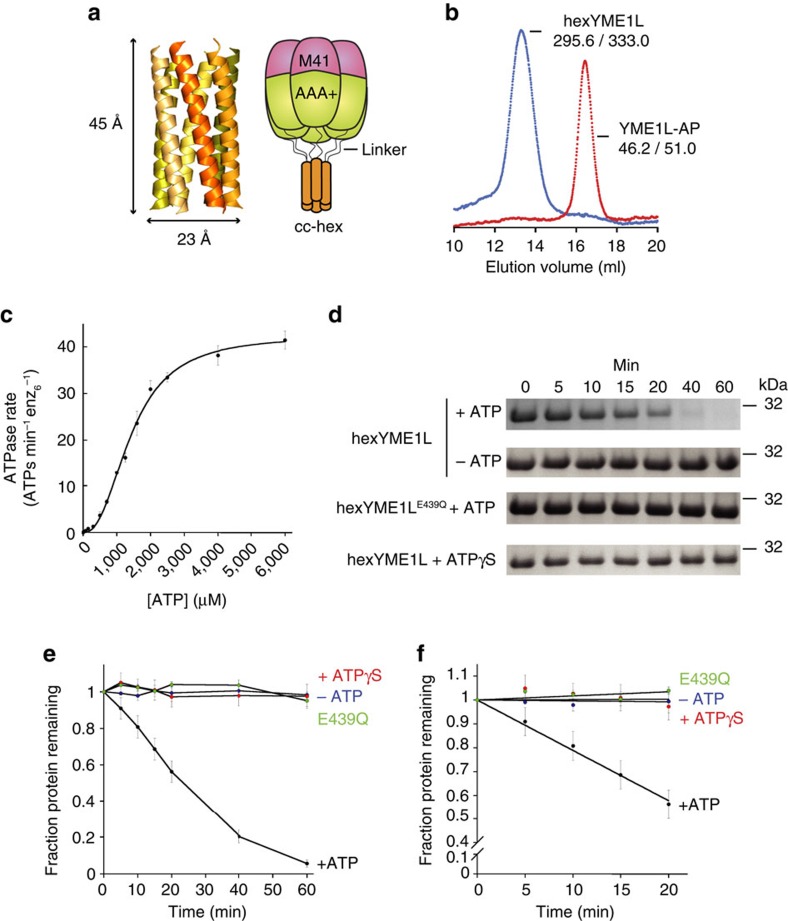
Engineering a soluble active YME1L protease. (**a**) Crystal structure of the cc-hex hexameric coiled coil (PDB ID: 3R3K) showing dimensions of the hexamer and schematic representation of the hexYME1L protease. (**b**) Migration profile of monomeric YME1L-AP and hexameric hexYME1L by size-exclusion chromatography showing observed and theoretical molecular weights for each species (observed/theoretical). (**c**) Rate of ATP hydrolysis by hexYME1L against increasing concentration of ATP. Lines are non-linear least-squares fits to the Hill version of the Michealis–Menten equation [*v*=*k*_ATPase_/(1+*K*_M_/[ATP]^n^] (*k*_ATPase_=42 ATPs min^−1^ enz_6_^−1^; *K*_M_=1.4 mM; *n*=2.6). (**d**) SDS–PAGE showing rapid degradation of β-casein (20 μM) by hexYME1L (1 μM). No degradation is observed in either the absence of ATP, the presence of the non-hydrolysable analogue ATPγS, or by a variant containing an ATPase abolishing mutation (hexYME1L^E439Q^) in the presence of ATP. (**e**) Loss of β-casein over time from degradation experiments in **d**. (**f**) Early time points (0–20 min) from **e**. Lines are linear fits used to calculate the initial rate of β-casein degradation by hexYME1L in the presence of ATP (0.49±0.08 molecules min^−1^ enz_6_^−1^). All data shown are from independent experiments and error bars indicate±s.e.m. (*n*=3).

Oligomerization of YME1L subunits through the transmembrane span is required to form the enzyme's ATPase sites and is necessary for ATP-dependent protein degradation. We sought to replace the insoluble transmembrane span with a soluble analogue capable of driving assembly of the AAA+ and protease domains to produce active hexameric proteases *in vitro*. We identified cc-hex, a 32-residue *de novo* designed peptide as a suitable candidate to replace the transmembrane span^33^. Multiple cc-hex peptides assemble in solution to form a hexameric coiled coil, with dimensions of ∼45 Å by ∼23 Å (Fig. 2a). Firstly, we identified a stable human YME1L construct containing the AAA+ ATPase and protease domains but lacking the N-terminal domain and transmembrane span (Fig. 1b; Supplementary Fig. 1). This catalytic core protein (YME1L-AP; residues 317 to 773) was expressed in *Escherichia coli*, purified to near homogeneity, and migrated by size exclusion chromatography (SEC) at a volume approximately corresponding to a monomer (apparent SEC molecular weight=46.2 kDa; calculated molecular weight=51.0 kDa) (Fig. 2b). Secondly, we genetically fused the cc-hex sequence to the N terminus of YME1L-AP separated by a ten-residue linker (GSGSYFQSNA). The purified fusion protein (hexYME1L) migrated approximately as a hexamer (SEC=295.6 kDa; calculated=333.0 kDa) (Fig. 2b). No change in migration was observed either in the presence of the non-hydrolysable ATP analogue, ATPγS, or after incubation with high concentrations of EDTA to remove co-purified nucleotide from the protein (Supplementary Fig. 2a).

ATP hydrolysis activity by YME1L requires the formation of a productive interface between subunits. To confirm that fusion of the catalytic core with cc-hex creates functional hexamers, we measured steady-state ATPase kinetics of the fusion protein using an established coupled-enzyme assay^34^. Purified hexYME1L displayed robust activity (*k*_ATPase_=42 ATPs min^−1^ enz^−1^; *K*_M_=1.4 mM; Fig. 2c), comparable to rates and affinities observed for solubilized FtsH and reconstituted 26S proteasomes^35^,^36^,^37^. The capacity of the enzyme to bind, translocate and degrade a substrate protein was demonstrated by monitoring the degradation of the model substrate β-casein in the presence of ATP (Fig. 2d–f; Supplementary Fig. 2b–e). No degradation was observed either in the absence of ATP or in the presence of ATPγS. To confirm that these activities do not result from contaminating enzymes, a hexYME1L variant was constructed bearing a mutation in the conserved Walker-B motif to abrogate ATPase activity (hexYME1L^E439Q^). This protein exhibited no observable ATPase or protein degradation activity. The initial rate of β-casein degradation by hexYME1L was calculated as 0.49±0.08 β-casein molecules min^−1^ enz_6_^−1^ (Fig. 2f). Taken together, these results confirm that hexYME1L is competent to carry out all of the basic activities of an energy-dependent protease and is suitable for *in vitro* studies.

### YME1L recognizes substrates via unstructured degrons

**Figure 3:**
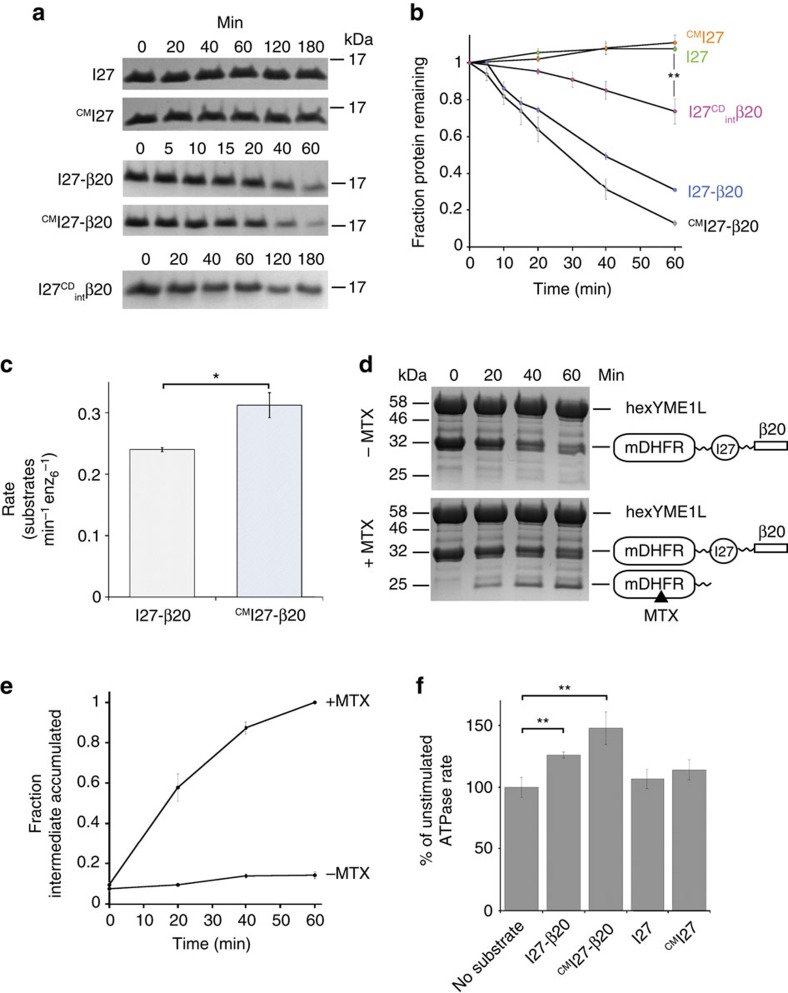
YME1L degrades substrates in a degron-dependent manner. (**a**) SDS–PAGE showing degradation of I27 variants (20 μM) by hexYME1L (1 μM). Rapid degradation is observed only for variants bearing the β20 degron. (**b**) Loss of I27 variants over time from degradation reactions in **a**. (**c**) Initial degradation rates of I27-β20 and ^CM^I27-β20 by hexYME1L calculated from linear fits to early time points (0–20 min) from **b**. (**d**) SDS–PAGE showing the processive degradation of mDHFR-I27-β20 (10 μM) by hexYME1L (2 μM) in the presence and absence of methotrexate (100 μM). Degradation in the presence of methotrexate results in the accumulation of a protected intermediate corresponding to the molecular weight of the mDHFR protein. (**e**) Accumulation of the intermediate in the presence and absence of methotrexate from experiments in **d**. (**f**) Relative stimulation of the ATPase rate of hexYME1L (0.25 μM) by the addition of I27 variants (20 μM). Significant stimulation is seen in the presence of folded I27-β20 (26%) and unfolded ^CM^I27-β20 (48%) but not in the presence of folded I27 (6%) or unfolded ^CM^I27 (14%). *P≤0.05, **P≤0.01 calculated from an unpaired Student's *t*-test (two-tailed). All data shown are from independent experiments and error bars indicate±s.e.m. (*n*=3).

Many proteins are recognized as substrates of AAA+ proteases by the presence of specific degron sequences^13^. Possible mechanisms of substrate selection by YME1L include recognition of specific degron sequences that become exposed after substrate destabilization or engagement of simple unstructured regions^23^,^38^. To first answer the question of whether simple unfolding is sufficient to initiate proteolysis by YME1L, we followed degradation of the I27 domain of human titin in both a natively folded (I27) and irreversibly unfolded yet soluble form produced by carboxymethylation of the internal cysteine residues (^CM^I27)^39^. Unfolding of the carboxymethylated I27 variants was confirmed by circular dichroism spectroscopy and no aggregation was observed by measuring light absorbance above 320 nm (ref. 40; Supplementary Fig. 3). Neither the folded nor the unfolded form of the protein was degraded by hexYME1L in the presence of saturating concentrations of ATP. However, fusion of the β20 sequence, a known AAA+ protease degron (QLRSLNGEWRFAWFPAPEAV)^41^, to the C terminus of I27 resulted in rapid ATP-dependent degradation of the protein in both folded (I27-β20) and unfolded forms (^CM^I27-β20) (Fig. 3a–c; Supplementary Fig. 4a–c). These results indicate that simple protein unfolding is not sufficient to initiate degradation by YME1L but that a degron of suitable sequence is required to present a protein as a potential substrate. Unsurprisingly, the initial degradation rate of unfolded ^CM^I27-β20 (0.31±0.020 molecules min^−1^ enz_6_^−1^) was greater than folded I27-β20 (0.24±0.003 molecules min^−1^ enz_6_^−1^) likely reflecting the additional time of substrate unfolding (Fig. 3c). To determine whether the degron is required to be located at a substrate terminus, we monitored degradation of a previously described I27 variant containing an internal β20 sequence (residues 19 to 38) and bearing substitution of cysteine residues to aspartic acid to induce unfolding without chemical modification (I27^CD^_int_β20)^41^. This protein was degraded significantly slower than either I27-β20 or ^CM^I27-β20 indicating that the location of the degron at an internal position in a substrate alters its degradation by hexYME1L (Fig. 3a,b; Supplementary Fig. 4d). Loss of hexYME1L was observed in extended degradation reactions but little or no loss was seen in shorter reactions in the presence of well-degraded substrates (for example, I27-β20) or when using hexYME1L^E439Q^, respectively. This implies autodegradation of the protease that can be outcompeted by the addition of a good substrate. We expect that autodegradation occurs through the engagement of nonnative residues in the linkers between cc-hex and the AAA+ domain. We quantified the loss of hexYME1L in reactions containing I27-β20 or ^CM^I27-β20 as less than 6% over 1 h and initial degradation rates were calculated only for reactions that displayed less than 10% loss of protease over time (Supplementary Fig. 5a).

The observed degradation of hexYME1L substrates could be explained by either tethering of the substrate to the protease surface by the degron followed by translocation and proteolysis of any accessible unstructured regions or by processive unfolding and degradation from C-terminal degron to the N terminus. To distinguish between these two models we adopted a strategy previously used to demonstrate processive degradation by FtsH and Lon^30^,^35^. Degradation of the chimeric fusion protein mDHFR-I27-β20 was carried out in the presence or absence of methotrexate, a small molecule that binds to mDHFR and prevents unfolding^35^. The observed accumulation of a protected intermediate as the degradation reaction proceeded in the presence of methotrexate demonstrated that this substrate is consumed by processive unfolding and translocation of its domains from the degron terminus (Fig. 3d,e; Supplementary Fig. 4e). Another indicator of processive translocation seen in many AAA+ enzymes is stimulation of the ATP hydrolysis rate upon interaction of the central pore loops with translocating substrate. Addition of substrate to high concentrations of protease (>1 μM) yielded no significant increase in ATPase rate but strong stimulation was observed at lower enzyme concentrations (Supplementary Fig. 5b). Addition of 20 μM ^CM^I27-β20 to a reaction containing 0.25 μM hexYME1L increased the ATPase rate per enzyme by 48%, whereas folded I27-β20 produced a lesser stimulation (26%) and both folded and unfolded I27 lacking a β20 degron produced no significant increase (Fig. 3f). The observed stimulation of ATPase rate at lower enzyme concentrations is likely due to reduced auto-degradation providing a stimulated rate in the absence of substrate. Together, these results further support both the requirement for a substrate to contain a degron and for processive translocation of substrate through the central pore.

### YME1L is capable of unfolding stable substrates

**Figure 4:**
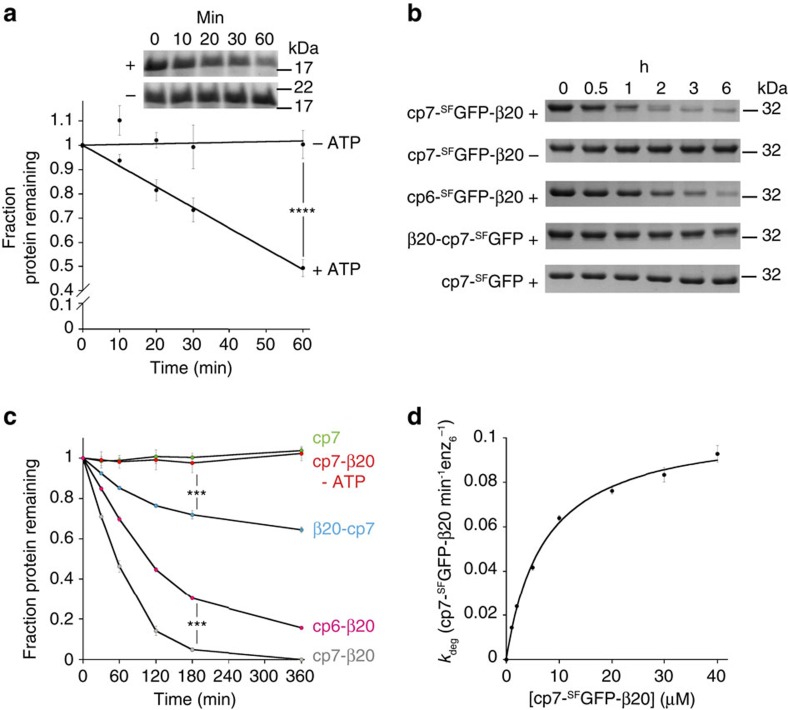
Unfolding and degradation of stable proteins by hexYME1L. (**a**) SDS–PAGE showing degradation of β20-λcI-N (20 μM) by hexYME1L (1 μM) in the presence (+) and absence (−) of ATP and a plot of the loss of β20-λcI-N over time. (**b**) SDS–PAGE showing degradation of circularly-permuted variants of GFP (20 μM) by hexYME1L (1 μM) in the presence (+) or absence (−) of ATP. (**c**) Loss of GFP variants over time from degradation experiments in **c**. (**d**) Rate of cp7-^SF^GFP-β20 degradation against increasing substrate concentration displaying a nonlinear least-squares fitting to the Michaelis–Menten equation (*k*_deg_=0.11 GFPs min^−1^ enz_6_^−1^; *K*_M_=7.1 μM). **P≤0.01, ***P≤0.001, ****P≤0.0001 calculated from an unpaired Student's *t*-test (two-tailed). All data shown are from independent experiments and error bars indicate±s.e.m. (*n*=3).

The observed degradation of folded I27 and mDHFR domains indicates that YME1L is capable of unfolding stable protein substrates before translocation and proteolysis. However, FtsH, a related bacterial membrane-anchored AAA+ protease, does not deliver a significant unfolding force and Yme1p has been shown to only degrade misassembled or destabilized mitochondrial components proteins^23^,^27^,^31^. To more clearly demonstrate whether YME1L can deliver a strong unfolding power stroke, we fused the β20 sequence to a number of proteins with known stabilities and examined their degradation by hexYME1L. The N-terminal domain of the λCI repressor protein (λCI-N) bearing a β20 degron at the N terminus was rapidly degraded by hexYME1L (Fig. 4a; Supplementary Fig. 6a). λCI-N is a stable protein (*T*_m_=54 °C) and present in >99% folded state at 37 °C (ref. 42), demonstrating that (i) YME1L is capable of unfolding a stable protein and (ii) can engage degrons from both N and C termini. We then monitored degradation of circularly permuted variants of GFP that exhibit altered unfolding pathways when degraded from the C terminus^43^ (Fig. 4b,c; Supplementary Fig. 6b–e). In all cases, hexYME1L was able to unfold and degrade these stable proteins. Substantial degradation was observed of both cp6-^SF^GFP-β20 (Δ*G*_unfolding_ of cp6-^SF^GFP=4.4 kcal mol^−1^) and cp7-^SF^GFP-β20 (Δ*G*_unfolding_ of cp7-^SF^GFP=4.9 kcal mol^−1^)^43^ (Fig. 4c). As with I27, the degradation of cp7-^SF^GFP-β20 was dependent on the presence of an unstructured β20 degron (Fig. 4c). A GFP variant bearing an N-terminal β20 tag (β20-cp7-^SF^GFP) underwent slow but observable degradation consistent with slower unfolding rates seen for other AAA+ proteases^43^ (Fig. 4c). Unfolding of the GFP β-barrel results in a measurable loss of fluorescence as the chromophore is exposed to solvent that correlates with protein degradation. To precisely quantify the substrate degradation activity of YME1L, we measured Michaelis–Menten kinetics of the degradation of cp7-^SF^GFP-β20, the fastest degraded fluorescent substrate (*k*_deg_=0.11 GFPs min^−1^ enz_6_^−1^; *K*_M_=7.1 μM (Fig. 4d; Table 1). The maximal degradation rate is approximately 7-fold slower than that observed for degradation of cp7-^SF^GFP bearing an alternative 20-residue C-terminal degron (cp7-^SF^GFP-Sul20; *k*_deg_=0.70 GFPs min^−1^ enz_6_^−1^; *K*_M_=2.8 μM) by *E. coli* Lon^44^. Although we cannot rule out contributions from the different degron sequences, the large difference in degradation rates under saturating substrate concentrations would suggest that YME1L is less proficient at unfolding cp7-^SF^GFP than Lon. This is further supported by the very slow observed degradation of β20-cp7-^SF^GFP by hexYME1L (Fig. 4b,c) compared with Lon (1.2 GFPs min^−1^ enz_6_^−1^)^44^. Thus, it appears that YME1L is competent to unfold and degrade highly stable proteins but with a significantly weaker unfolding power than robust unfoldases such as Lon and ClpX. The observed preference for degrading cp7-^SF^GFP bearing a C-terminal rather than N-terminal degron likely reflects a difference in the kinetics of unfolding of this substrate from either terminus. Indeed, differences in the degradation rates of proteins from different termini is a feature of other AAA+ proteases^30^.

### Degron sequence affects degradation by YME1L

**Figure 5:**
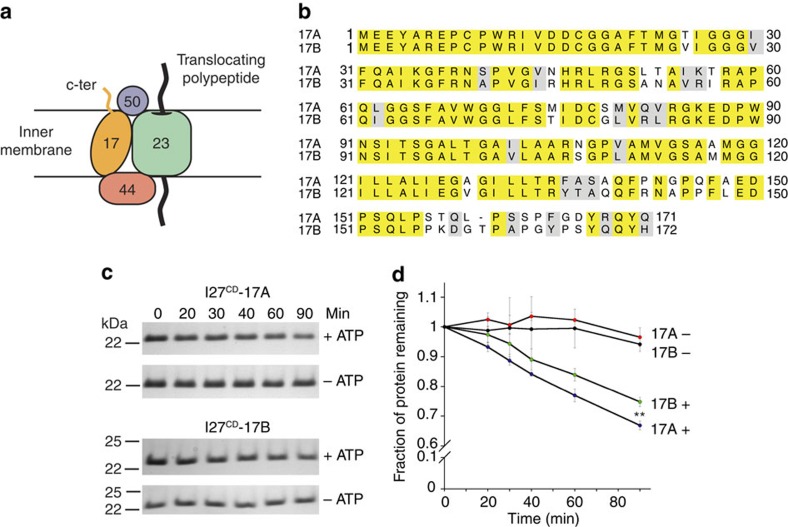
The C termini of TIM17A and TIM17B can promote degradation. (**a**) Cartoon showing organization of the TIM23 translocase complex. Known subunits of the complex include TIM23 (green), TIM50 (blue), TIM44 (red) and TIM17 (orange). The C-terminal tail of TIM17 is predicted to project into the intermembrane space. (**b**) Protein sequence alignment of human TIM17A and TIM17B. Identical aligned residues are coloured yellow and conservative substitutions are coloured grey. (**c**) SDS–PAGE showing degradation of I27^CD^-17A and I27^CD^-17B (20 μM) by hexYME1L (1 μM) in the presence or absence of ATP. (**d**) Loss of I27-Tim17 variants over time from degradation experiments in **c**. **P≤0.01 calculated from an unpaired Student's *t*-test (two-tailed). All data shown are from independent experiments and error bars indicate±s.e.m. (*n*=3).

The requirement for unfolded I27 to present an accessible β20 sequence to initiate degradation by hexYME1L implies that the protease can discriminate between accessible sequences when selecting substrates. To determine whether such selection can be observed in physiological substrates of YME1L, we examined the degradation of proteins containing sequences from two homologues of the human TIM17 protein: TIM17A and TIM17B. TIM17A and TIM17B are differentially expressed subunits of the TIM23 complex that translocates proteins across the mitochondrial inner membrane^45^ (Fig. 5a). Previously, TIM17A but not TIM17B has been shown to be degraded in a YME1L-dependent manner in response to mitochondrial stress^38^. Sequence alignments show that variation between the two homologous subunits largely occurs at the C termini, which are predicted to project into the intermembrane space^46^,^47^ (Fig. 5b). We reasoned that these C-terminal regions could act as an accessible degron for recognition by YME1L. Unfolded I27^CD^ fused to the C termini of TIM17A (I27^CD^-17A; 36 residues) and TIM17B (I27^CD^-17B; 37 residues) exhibited slow but measurable degradation by hexYME1L in the presence of ATP (Fig. 5c,d; Supplementary Fig. 7). The loss of I27^CD^-17A occurred significantly more rapidly than I27^CD^–17B but the difference in degradation rates did not reflect the degree of difference observed *in vivo* under stress conditions^38^ (Fig. 5d). These results suggest that the C-terminal tails of the TIM17 homologues may act as recognition sequences for YME1L but that the mechanism inducing TIM17A degradation under stress involves additional factors, potentially stress-dependent changes in protein stability or accessibility of the degron.

**Figure 6:**
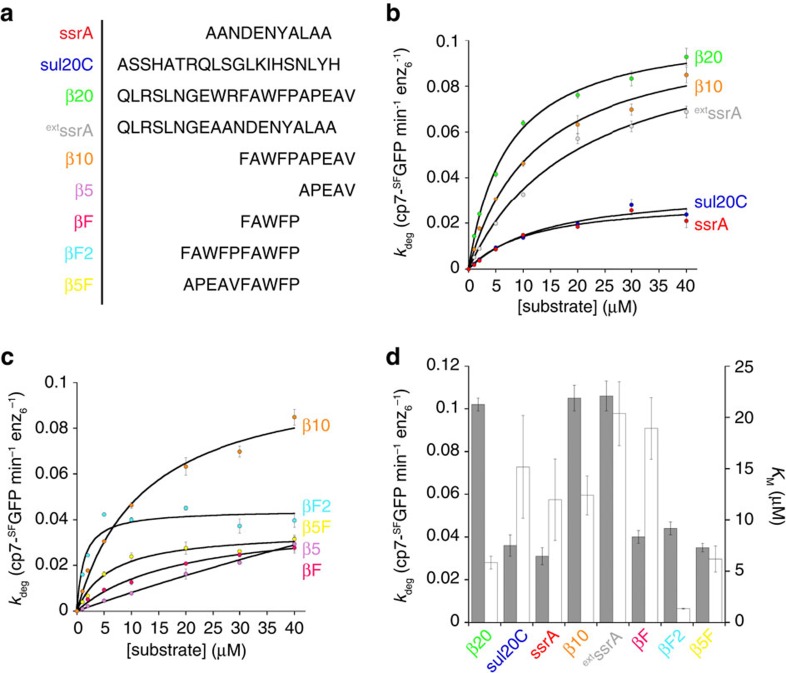
YME1L discriminates between degrons by sequence. (**a**) Sequences of each degron fused to cp7-^SF^GFP and tested for ATP-dependent degradation by hexYME1L. (**b**) Michaelis–Menten plots showing degradation of cp7-^SF^GFP variants bearing C-terminal fusions of degrons previously shown to enhance degradation by AAA+ proteases (β20 (green) and sul20C (blue), Lon protease; ssrA (red), ClpXP protease; β10 (orange); ^ext^ssrA (grey)). Lines are nonlinear least-squares fits to the Michealis–Menten equation. (**c**) Michaelis–Menten plots of cp7-^SF^GFP proteins bearing C-terminal fusions of multiple truncated variants of the β20 degron (β10 (orange); β5, (pink); βF (magenta); βF2 (cyan); β5F (yellow)). Lines are nonlinear least-squares fits to the Michealis–Menten equation. (**d**) Maximal degradation rates (black columns) and K_M_ values (white columns) for each degron sequence fused to the C-terminus of cp7-^SF^GFP. All degradation reactions contained hexYME1L (1 μM). All data shown are from independent experiments and error bars indicate±s.e.m. (*n*=3).

To provide further evidence that YME1L can select substrates on the basis of sequence, we tested a series of known degron that target proteins to other AAA+ proteases (Table 1; Fig. 6a). Each degron was fused to the C terminus of cp7-^SF^GFP, the fastest degraded fluorescent protein, to prevent slow unfolding from masking differences in recognition. The highest rate of degradation was observed for cp7-^SF^GFP-β20. Slower degradation was seen for the 20-residue sul20C tag (ASSHATRQLSGLKIHSNLYH; *k*_deg_=0.036±0.005 GFPs min^−1^ enz_6_^−1^; *K*_M_=15.2±5.0 μM), another known *E. coli* Lon degron, and the 11-residue ssrA tag (AANDENYALAA; *k*_deg_=0.031±0.004 GFPs min^−1^ enz_6_^−1^; *K*_M_=12.0±4.0 μM), a known *E. coli* ClpXP degron (Fig. 6b,d). The lower maximal degradation rate of cp7-^SF^GFP-sul20C compared with cp7-^SF^GFP-β20, two degrons of equal length but different sequence, demonstrated that YME1L can discriminate between degrons on the basis of sequence composition. The slow degradation of cp7-^SF^GFP-ssrA could be explained either by a poorly recognized sequence preventing substrate binding or a shorter tag length preventing proper engagement with the translocation machinery in the central pore. Removal of ten N-terminal residues from the β20 sequence to leave a ten-residue tag (cp7-^SF^GFP-β10; FAWFPAPEAV) had no measurable effect on *k*_deg_ when compared with β20 and only increased *K*_M_ of the reaction approximately two-fold (*k*_deg_=0.105±0.015 GFPs min^−1^ enz_6_^−1^; *K*_M_=12.4±1.9 μM). This suggests that the N-terminal portion of the tag is not significantly involved in recognition by YME1L and that a 10-residue tag can be effectively engaged. However, a 20-residue extended ssrA degron containing the N-terminal nine residues of the β20 sequence (cp7-^SF^GFP-^ext^ssrA; QLRSLNGEAANDENYALAA) was degraded significantly more quickly than cp7-^SF^GFP-ssrA but with a higher K_M_ than β20 (*k*_deg_=0.106±0.007 GFPs min^−1^ enz_6_^−1^; *K*_M_=20.4±3.1 μM) (Fig. 6b,d). Together, these results suggest that YME1L uses degron sequences to discriminate between substrates but that the length of the degron also contributes significantly to the kinetics of degradation by the protease.

The higher rate of degradation observed for β20 tagged substrates compared with other potential degrons presented this sequence as the most suitable for further analysis to discover the contributions of degron sequence to substrate recognition by YME1L. We produced a series of additional cp7-^SF^GFP constructs bearing shortened variants of the β20 tag (Fig. 6a). Truncation to the C-terminal five residues of β20 (cp7-^SF^GFP-β5; APEAV) dramatically lowered the affinity of the enzyme for this substrate, preventing saturation and making accurate determinations of *k*_deg_ and *K*_M_ impossible. These results suggest that either the protease requires greater than five accessible amino acids to engage substrates or that the primary recognition sequence lies in the portion removed between the β10 and β5 tags. A cp7-^SF^GFP fusion bearing these five removed residues (cp7-^SF^GFP-βF; FAWFP) recovered moderate degradation (*k*_deg_=0.040±0.003 GFPs min^−1^ enz_6_^−1^; *K*_M_=18.9±3.0 μM) (Fig. 6c,d). Together, these results indicate that FAWFP is likely the primary recognition motif in the β20 degron for YME1L although additional residues in the degron are required for efficient degradation. A repeat FAWFP motif degron (cp7-^SF^GFP-βF2; FAWFPFAWFP) displayed a greater affinity than any other degron but did not increase the maximal degradation rate (*k*_deg_=0.044±0.003 GFPs min^−1^ enz_6_^−1^; *K*_M_=1.3±0.4 μM). One explanation for this observation is that the presence of two FAWFP motifs increases the strength of the interaction between enzyme and substrate preventing efficient translocation through the central pore. To investigate whether the position of the FAWFP motif in the degron impacts substrate recognition, we tested degradation of a reordered β10 sequence with the FAWFP motif located at the very C terminus (cp7-^SF^GFP-β5F; APEAVFAWFP). The maximal degradation rate for this substrate was significantly lower than β10 and similar to that of βF (*k*_deg_=0.035±0.002 GFPs min^−1^ enz_6_^−1^; *K*_M_=6.2±1.3 μM), indicating that although the FAWFP motif is important for recognition, placing the motif at the very C terminus in either a five or ten-residue degron results in reduced substrate processing.

## Discussion

The mitochondrial AAA+ proteases are anchored into the inner membrane by insoluble transmembrane spans. The successful replacement of the N-terminal domain and transmembrane span of YME1L with a soluble peptide bearing no sequence homology demonstrates that these domains are not required to actively participate in substrate binding, destabilization or translocation. Instead, these regions likely provide the interactions to assemble the YME1L subunits into hexamers and insert the enzyme into the site of action. However, we cannot rule out the possibility that the transmembrane spans play a role in binding or processing of a subset of the physiological substrates of YME1L, most likely integral inner membrane proteins.

Our results demonstrate that the presence of an unstructured degron at the terminus of a protein can drive degradation by YME1L. However, it is also clear from the degradation kinetics of proteins bearing different terminal sequences that YME1L can discriminate between substrates on the basis of sequence composition. The previously observed degradation of thermally destabilized proteins by Yme1p in yeast mitochondria could be explained by the inability of the protease to unfold stable domains or by the appearance of degron sequences upon unfolding^23^. Given the observed unfolding of stable proteins such as cp7-^SF^GFP, we conclude that destabilization drives the appearance of sequences that can be recognized by YME1L rather than simply removing the requirement for unfolding. Our results suggest the following general principles of substrate recognition by YME1L: (i) substrates must contain an accessible unstructured degron that may be as short as five amino acids and (ii) the identity of the degron sequence is more important than the length of the unstructured region. This model is broadly consistent with *in vivo* experiments that suggest the mitochondrial AAA+ proteases require an accessible sequence of approximately 10–20 residues^23^. The engagement of a shorter unstructured degron by hexYME1L than seen *in vivo* could be explained by the interaction of two freely moving soluble proteins versus substrate binding to an enzyme fixed in the inner membrane.

We have identified a primary targeting sequence (FAWFP) within the β20 sequence that appears to be responsible for recognition by the protease. Interestingly, a similar sequence (WRFAWFP) was previously identified as the primary binding site of the β20 degron to *E. coli* Lon^41^. Moreover, a similar hydrophobic motif (FPLF) was shown to target the DNA damage repair protein UmuD to Lon^48^. Parallels in recognition sequences between human YME1L and *E. coli* Lon, two distantly related proteases, may signify that the enzymes have similar substrate profiles. Lon acts as a general housekeeping protease, binding hydrophobic regions that become exposed upon substrate unfolding, and also regulates levels of specific cellular proteins by recognition of defined degron sequences^41^,^49^. Given the roles that YME1L appears to play in both the removal of both misassembled proteins and specific intermembrane space components, it is possible that these two proteases employ similar modes of substrate recognition. We hypothesized that motifs similar to FAWFP may be found in known YME1L substrates. A search of the mitochondrial IMS proteome did not yield any proteins containing the motif FAWFP^50^,^51^. However, the motif F-h-h-F (h=hydrophobic) was identified in 21 of the 127 listed human IMS proteins (Supplementary Table 1), including two confirmed substrates of YME1L: the inner membrane protease OMA1 (ref. 52; FVVF) at a position in a predicted transmembrane region; and the N terminus of the lipid carrier protein PRLD1 (FAAF)^53^.

Variants of the β20 degron presenting the FAWFP motif at the far C terminus exhibited high affinity for the protease but lower degradation rates, indicating defects in substrate engagement. It is possible that the addition of residues with low affinity for protease (for example, APEAV) could relieve these defects. The substrate binding sites of Yme1p have been broadly mapped by truncation experiments to two regions at the N and C termini of each subunit, some distance from the central pore^32^. The presence of a number of low-affinity residues at the terminus of the degron may allow simultaneous binding of the FAWFP motif and engagement of the protein terminus with the machinery of translocation and enable efficient degradation.

The experiments described here give numerous examples of the degradation of stable folded proteins including mDHFR, the I27 domain of titin, and variants of GFP. The circularly permuted GFP variants in particular have measured thermodynamic stabilities of several kcal mol^−1^ and thus their degradation strongly implies that YME1L is capable of delivering a significant unfolding force. The maximal degradation rate of ^SF^GFP-cp7-β20 was ∼10-fold slower than seen for the same variant fused to a C-terminal ssrA tag by *E. coli* ClpXP and ∼7-fold slower than proteolysis of a C-terminal sul20C tagged variant by *E. coli* Lon^43^,^44^. In contrast, the best-studied membrane-anchored AAA+ protease, *E. coli* FtsH, cannot unfold and degrade either GFP or unmodified mDHFR. Koodathingal and co-workers defined a processivity ratio *U* to describe unfolding by a AAA+ protease based on the relative accumulation of a mDFHR fusion intermediate in the presence and absence of methotrexate^30^,^35^. Using this approach we calculated a ratio of ∼6 for the degradation of mDHFR-I27-β20 by hexYME1L-AP, signifying a predominance of successful mDHFR unfolding events over dissociation of the partially degraded intermediate from the protease. This compares to a value of ≥10 for unfolding of the same substrate by *E. coli* Lon and ∼3 for Lon unfolding mDHFR-I27 fusion bearing an N-terminal β20 degron^30^.

By precisely measuring the rates of ATP hydrolysis and protein degradation by hexYME1L-AP we can calculate the efficiency of degradation to be ∼400 ATPs consumed per ^SF^GFP-cp7-β20 molecule degraded. This value is significantly higher than that observed for degradation of cp7-^SF^GFP fused to the sul20C degron by *E. coli* Lon (∼190 ATPs per ^SF^GFP-sul20C degraded^44^). Although differences in degradation efficiency could be partially explained by interaction of the proteases with different degrons, YME1L appears to be much less efficient at converting ATP into protein degradation. This difference is more likely explained by the weaker unfolding power of YME1L leading to a greater number of nonproductive ATP hydrolysis events as the substrate resists unfolding. In conclusion, we propose that YME1L occupies an intermediate position in a ranking of AAA+ proteases by unfolding power between the strong unfoldases such as ClpX, ClpA and Lon, and enzymes that lack significant unfolding power such as FtsH. Interestingly, the *S. cerevisiae* YME1L homologue, Yme1p, degrades the small intermembrane space translocase proteins, Tim9 and Tim10 after destabilization of the substrate's structure by disruption of internal disulfide bonds^25^. A similar weak power stroke to that observed for the human protease could prevent translocation of the disulfide-bonded species and provide a means of discriminating between the functional and nonfunctional forms of these chaperones. Indeed, a similar mechanism for a AAA+ protease to use low unfolding power to selectively degrade destabilized proteins has been proposed for FtsH^31^.

The AAA+ family of enzymes regulate diverse cellular processes including transcription, secretion, and the movement of intracellular cargo^54^. Elucidating the mechanisms that these enzymes use to couple ATP binding and hydrolysis to the creation of force requires detailed measurements in isolation from other cellular components. These activities have been well studied in the case of many soluble AAA+ enzymes, including ClpX^28^, dynein^55^ and numerous helicases^56^,^57^. However, difficulties in producing active membrane-anchored AAA+ enzymes have hampered our understanding of important enzymes that operate at the interface of membrane and solution environments. We have overcome this obstacle by providing the interactions needed to assemble an active AAA+ enzyme from a soluble oligomerization unit. Given that proteolysis by YME1L requires formed ATPase active sites between subunits, it is clear that fusion to the cc-hex coiled-coil allows for correct assembly of the hexamer. The possibility remains that the coiled-coil impedes movement of YME1L domains during the catalytic cycle and thus limits degradation activity. However, the rate of ATP hydrolysis observed for hexYME1L-AP is similar to that measured for solubilized *E. coli* FtsH containing its native transmembrane structure^35^. Moreover, models constructed using crystal structures of FtsH and sequence prediction of the YME1L transmembrane domain suggest that the structured cc-hex and AAA+ domains in hexYME1L-AP are separated by a greater number of flexible unstructured residues than the transmembrane and AAA+ domains of native YME1L. We expect that this approach to hexamer assembly may be applied to enable *in vitro* analysis of the activities of other membrane AAA+ enzymes, such as spastin and katanin^58^,^59^.

## Methods

### Cloning and construct design

To produce monomeric catalytic domains of YME1L (YME1L-AP) a sequence encoding residues 317–773 of human YME1L isoform 1 (ExPASy ID: Q96TA2) was amplified by PCR and inserted into the 2G-T ligation-independent cloning vector (Addgene ID: 29707) containing an N-terminal His_6_-GST tag. Hexameric YME1L (hexYME1L) was produced by inserting a 32-residue codon optimized cc-hex sequence (GELKAIAQELKAIAKELKAIAWELKAIAQGAG; Genscript) directly N-terminal of the YME1L-AP coding sequence, separated by a 10-residue linker sequence (GSGSYFQSNA). The hexYME1L^E439Q^ variant was produced by site directed mutagenesis using the hexYME1L plasmid as a template. Plasmids containing the cp7-^SF^GFP, cp6-^SF^GFP (ref. 44), I27, I27-β20, I27^CD^_int_β20 (ref. 41), mDHFR-sul20 (ref. 30) and β20-λCI-N (ref. 41) protein coding sequences were a generous gift from Prof. Robert Sauer (MIT) and appropriate degron sequences were added to either the N or C termini by PCR. The mDHFR-I27-β20 chimeric fusion was constructed from a mDHFR-sul20C template by first replacing the sul20C coding sequence with that of β20 by PCR. The I27 coding sequence was inserted between mDHFR and β20 by PCR with a four-residue linker separating mDHFR and I27. Plasmids encoding the C-terminal sequences of human TIM17A (ExPASy ID: Q99595; residues 136 to 171) and TIM17B (ExPASy ID: O60830; residues 136 to 172) were a gift from Dr R. L. Wiseman (TSRI). The I27^CD^-17A and I27^CD^-17B proteins were produced by appending these encoding sequences directly to the C-terminus of the I27^CD^ protein by PCR.

### Protein expression and purification

*E. coli* BL21 (DE3) cells with plasmids containing YME1L-AP or hexYME1L were grown at 37 °C to OD_600_=0.3, followed by growth at 16 °C to OD_600_=0.6. Protein expression was induced by addition of 0.5 mM IPTG at 16 °C for 16 h. Cells were harvested and lysed by sonication in a buffer containing 25 mM Tris HCl (pH 8.0), 300 mM NaCl, 10% glycerol, 0.1 mM EDTA, 10 mM MgCl_2_, 10 mM β-mercaptoethanol and 1 mM PMSF. Cell lysate was clarified by centrifugation and applied to a column containing Glutathione Superflow Agarose (Pierce). Unbound proteins were removed by washing with lysis buffer lacking PMSF and target proteins eluted by addition of 10 ml of lysis buffer supplemented with 10 mM glutathione. The His_6_-GST tag was removed by incubation of the eluted protein with 1 mg TEV protease per 50 mg of fusion protein at 4 °C overnight followed by removal of the cleaved tag and TEV protein by binding to Ni-NTA agarose (Thermo Scientific). Further purification was carried out by SEC using a Superose 6 10/300 GL column (GE Healthcare) equilibrated with buffer containing 10 mM Tris HCl (pH 8.0), 100 mM NaCl, 10% glycerol, 0.1 mM EDTA, 5 mM MgCl_2_ and 1 mM dithiothreitol (DTT). Fractions containing the target protein that migrated at a volume corresponding to the correct oligomeric state were pooled, concentrated and flash-frozen in liquid nitrogen before storage at −80 °C.

All circularly permuted GFP variants and mDHFR-I27-β20 were grown in *E. coli* BL21 (DE3) at 37 °C to OD_600_=0.6 and expressed by induction with 1 mM IPTG at 16 °C for 16 h. Cells were pelleted and re-suspended in buffer containing 25 mM Tris HCl (pH 8.0), 300 mM NaCl, 10% glycerol, 10 mM βME and 10 mM imidazole followed by lysis by sonication and clarified by centrifugation. Initial purification was carried out by addition of the supernatant to a gravity column containing Ni-NTA agarose resin (Thermo Scientific). Fractions containing target proteins were pooled and further purified by SEC using a HiLoad 16/600 Superdex 200 column (GE Healthcare) pre-equilibrated with buffer containing 10 mM Tris HCl (pH 8.0), 100 mM NaCl, 10% glycerol and 1 mM DTT. Fractions containing target proteins were pooled, concentrated, flash-frozen in liquid nitrogen and stored at −80 °C.

Plasmids containing I27 variants were grown in *E. coli* BL21 (DE3) cells at 37 °C to OD_600_=0.6 followed by addition of 0.5 mM IPTG to induce protein expression for 16 h at 18 °C. Harvested cells were re-suspended in a buffer containing 20 mM Tris HCl (pH 8.0), 300 mM NaCl, 10 mM imidazole, 10% glycerol and 1 mM PMSF and lysed by sonication. Cell lysate was clarified by centrifugation and the supernatant was applied to a Ni-NTA agarose resin (Thermo Scientific) followed by washing with lysis buffer supplemented with 50 mM imidazole. Bound proteins were eluted in lysis buffer supplemented with 500 mM imidazole. Fractions containing target protein were pooled and applied to a HiLoad 16/600 Superdex 200 column (GE Healthcare) pre-equilibrated with buffer containing 10 mM Tris HCl (pH 8.0), 100 mM NaCl and 10% glycerol. I27^CD^_int_β20, I27^CD^-17A, and I27^CD^-17B were expressed similarly to the soluble I27 variants except that expression was carried out at 37 °C for 3 h. Cells were lysed in a buffer containing 20 mM Tris HCl (pH 8.0). After centrifugation at 15,000 r.p.m. for 30 min the pellet was washed twice with lysis buffer and solubilized with 15 ml of lysis buffer supplemented with 6 M guanidine hydrochloride (GuHCl). Solubilized proteins were bound to Ni-NTA agarose resin followed and unbound proteins washed off in lysis buffer supplemented with 10 mM imidazole. Target proteins were eluted in lysis buffer supplemented with 500 mM imidazole and then buffer exchanged into 10 mM Tris HCl (pH 8.0), 100 mM NaCl using a HiTrap Desalting column (GE Healthcare). All I27 variant proteins were concentrated and flash-frozen in liquid nitrogen before storage at −80 °C (ref. 41).

### Protein carboxymethylation

Carboxymethylation of cysteine residues in I27 variants was carried out by unfolding proteins in a buffer containing 0.6 M Tris-HCl (pH 8.6), 6 M GuHCl and 5 mM DTT for 3 h at 25 °C (ref. 39). Hundredfold molar ratio of freshly prepared iodoacetic acid was then added for 2 h in the dark. After carboxymethylation, proteins were buffer exchanged into 5 mM sodium phosphate (pH 8.0) using a HiTrap Desalting column (GE Healthcare).

### Spectroscopy

Circular dichorism spectra were measured using an Olis RSM CD spectrophotometer at 25 °C with a protein concentration of 20 μM and a path length of 1 mm. Raw elipticity values were measured between 310 nm to 190 nm with a step size of 0.5 nm and converted to molar elipticity (degs cm^2^ mol^−1^). UV/visible light absorbance spectra were measured at 37 °C between 200 nm and 550 nm using a SpectraMax M5 plate reader in a quartz cuvette with path length of 1 cm, step size of 1 nm and a protein concentration of 100 μM.

### Analytical size exclusion chromatography

All analytical size exclusion chromatography experiments were carried out by injecting 1 mg of protein in a volume of 200 μl on to a Superose 6 Increase 10/300 GL column (GE Healthcare). Samples without added nucleotide or EDTA were incubated on ice for 1 h before injection onto a column equilibrated with buffer A (50 mM Tris-HCl (pH 8.0), 100 mM NaCl and 0.5 mM TCEP). EDTA-treated samples were incubated with 5 mM EDTA on ice for 1 h then loaded to a column pre-equilibrated with buffer A supplemented with 5 mM EDTA. ATPγS treated samples were incubated with 10 mM ATPγS and 10 mM MgCl_2_ for 1 h on ice and then loaded to a column equilibrated with buffer A supplemented with 100 μM ATPγS and 100 μM MgCl_2_.

### ATPase assays

ATPase assays were conducted using a coupled-enzyme assay monitoring the loss of NADH. Reactions were carried out at 37 °C in a buffer containing 25 mM HEPES-KOH (pH 8.0), 10% glycerol, 5 mM MgCl_2,_ 1 μM hexYME1L, 1 mM NADH, 21.5 U ml^−1^ lactate dehydrogenase and an ATP regeneration system (5 mM ATP, 7.5 mM phosphoenolpyruvate (PEP) and 18.8 U ml^−1^ pyruvate kinase (PK)). Loss of NADH absorbance at 340 nm was measured in a 384-well plate using a SpectraMax M5 plate reader (Molecular Devices). Stimulation of ATP hydrolysis was induced by addition of 20 μM substrate to each reaction.

### Protein degradation assays

All degradation assays were carried out at 37 °C using 1 μM or 2 μM hexYME1L-AP in buffer containing 25 mM HEPES-KOH (pH 8.0), 100 mM KCl, 10 mM MgCl_2_, 1 mM DTT, 10% glycerol, 25 μM ZnCl_2_ and an ATP regeneration system (5 mM ATP, 20 mM PEP and 18.75 U ml^−1^ PK). Time-course degradation reactions assessed by SDS–PAGE contained 20 μM substrate in a total volume of 100 μl and aliquots were quenched in SDS–PAGE loading buffer containing 2% SDS at 90 °C. Reactions were supplemented with 0.1 mg ml^−1^ creatine kinase as a loading control. All SDS–PAGE bands were visualized by staining with Coomassie Brilliant Blue R-250 (Bio-Rad). Substrate band intensities were quantified using ImageJ^60^ and normalized to the creatine kinase loading control. The positions of molecular weight markers are marked on cropped SDS–PAGE images and un-cropped images for all SDS–PAGE experiments are presented in the Supplementary Material. Degradation kinetic measurements following loss of fluorescence were measured in 30 μl reactions in a 384-well plate using a SpectraMax M5 plate reader (excitation=467 nm; emission=511 nm). Degradation of 10 μM mDHFR-I27-β20 was carried out in the presence and absence of 100 μM methotrexate. The processivity ratio is defined as [*U*=*k*_deg_/*k*_rel_=(*I*_+mtx_/*I*_−mtx_)−1] where *k*_deg_ is the rate of successful unfolding, *k*_rel_ is the rate of substrate release, and *I*_+mtx_ and *I*_−mtx_ are the intensities of partially degraded products observed by SDS–PAGE in the presence and absence of methotrextate, respectively. Intensities of SDS–PAGE bands corresponding to the partially degraded intermediates were quantified using ImageJ^60^ and normalized to the intensity of the protease band as a loading control.

### Data availability

The data that support the findings of these studies are available from the corresponding author upon reasonable request.

## Additional information

**How to cite this article:** Shi, H. *et al*. Engineered AAA+ proteases reveal principles of proteolysis at the mitochondrial inner membrane. *Nat. Commun.* **7,** 13301 doi: 10.1038/ncomms13301 (2016).

**Publisher's note:** Springer Nature remains neutral with regard to jurisdictional claims in published maps and institutional affiliations.

## Supplementary Material

Supplementary Information Supplementary Figures 1-7 and Supplementary Table 1

## Figures and Tables

**Table 1:** Degradation of GFP proteins bearing different degron sequences.

Degron	Number of residues	Sequence	*k*_deg_ (min^−1^enz_6_^−1^)	*K*_M_ (μM)
β20	20	QLRSLNGEWRFAWFPAPEAV	0.110±0.003	7.1±0.6
ssrA	11	AANDENYALAA	0.031±0.004	12.0±4.0
sul20C	20	ASSHATRQLSGLKIHSNLYH	0.036±0.005	15.2±5.0
β10	10	FAWFPAPEAV	0.105±0.015	12.4±1.9
^ext^ssrA	19	QLRSLNGEAANDENYALAA	0.106±0.007	20.4±3.1
β5	5	APEAV	ND	ND
βF	5	FAWFP	0.040±0.003	18.9±3.0
βF2	10	FAWFPFAWFP	0.044±0.003	1.3±0.4
β5F	10	APEAVFAWFP	0.035±0.002	6.1±0.6

ND, not determined.
