# Supplementary material for: Engineered AAA+ proteases reveal principles of proteolysis at the mitochondrial inner membrane
Source: Nat Commun. 2016 Oct 27;7:13301. doi: 10.1038/ncomms13301 (PMC5095350; doi:10.1038/ncomms13301)
Supplement: Supplementary Information — Supplementary Figures 1-7 and Supplementary Table 1 [file ncomms13301-s1.pdf]

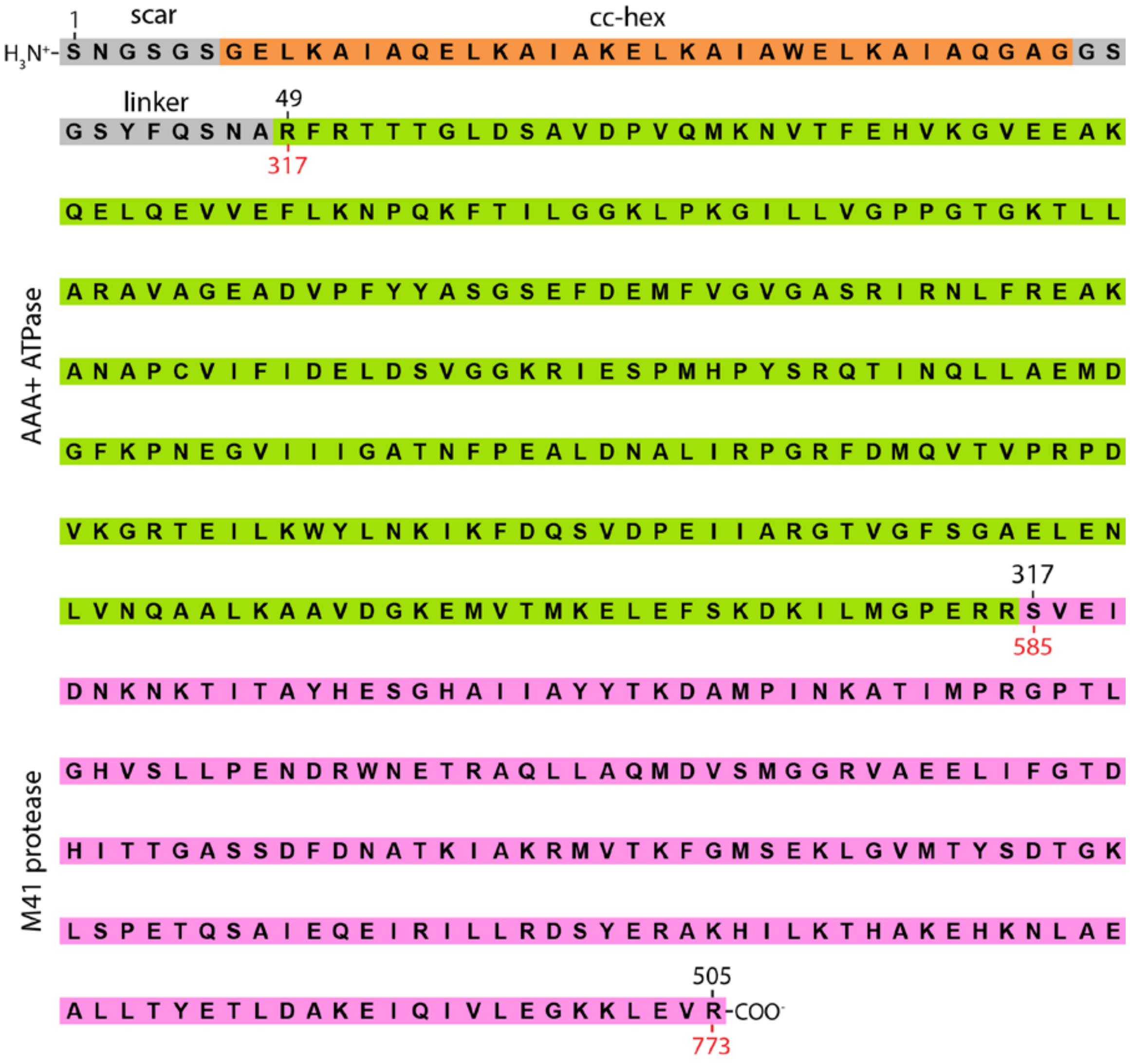

Supplementary Figure 1. Protein sequence map of hexYME1L.
Amino acid sequence of the hexYME1L construct showing the positions of the cc-hex sequence (orange), AAA+ ATPase domain (green), protease domain (pink) and linkers and scar produced after TEV cleavage (gray). Residue numbers correspond to the hexYME1L sequence (black) or the native YME1L protein sequence (red).

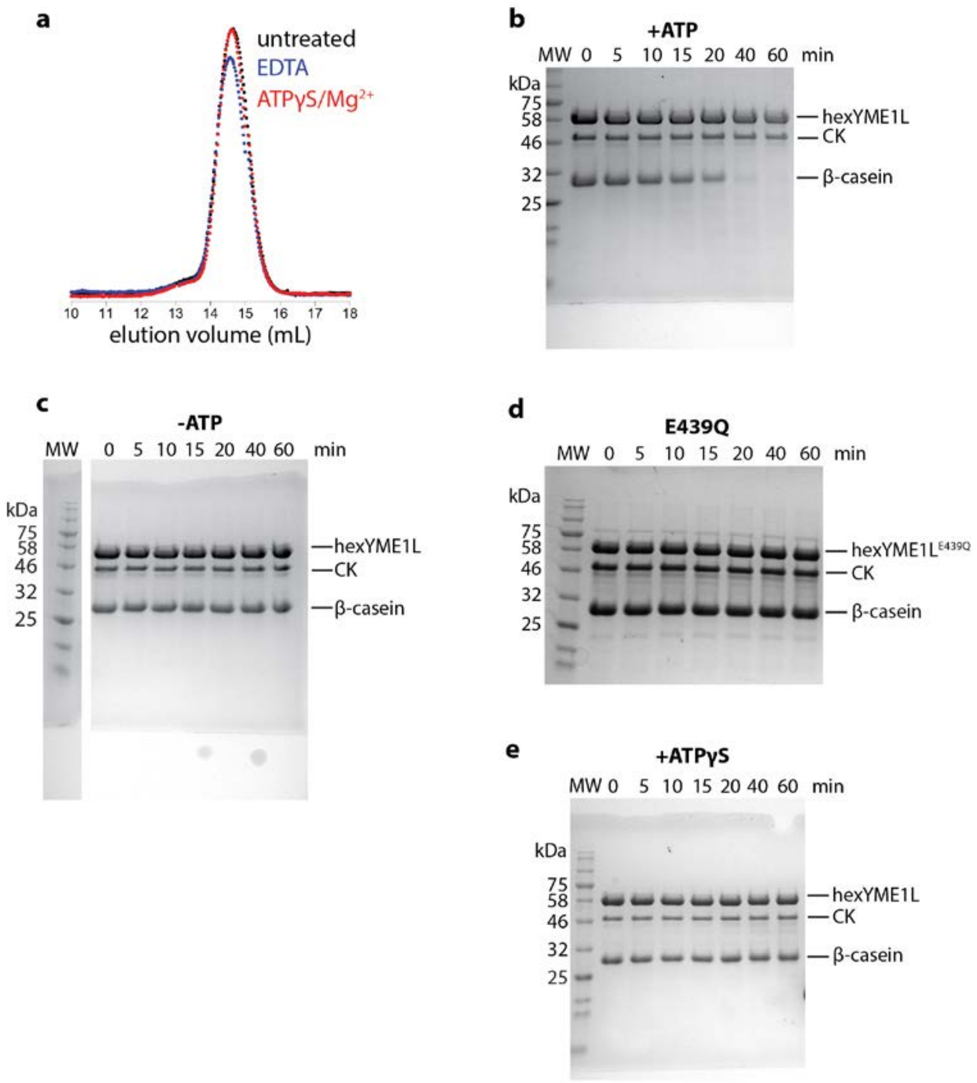

Supplementary Figure 2. An engineered YME1L protease is active for degradation. (a) Migration profile of hexYME1L by size-exclusion chromatography without additional treatment (black); after incubation with 5 mM EDTA to remove co-purified nucleotide (blue); in the presence of 10 mM ATPγS (red). (b-e) Full SDS-PAGE images for experiments in Fig. 2: (b) hexYME1L in the presence of ATP; (c) hexYME1L in the absence of ATP; (d) hexYME1L^{E439Q} in the presence of ATP; (e) hexYME1L in the presence of ATPγS (5 mM). All degradation reactions contained 0.1 mg ml^{-1} creatine kinase (CK) as a loading control.

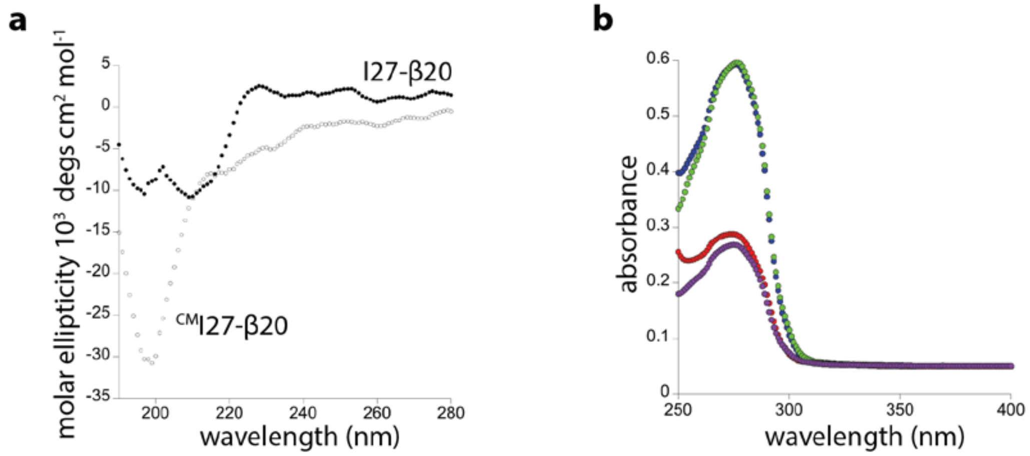

Supplementary Figure 3. Spectroscopy of I27 variants. (a) Circular dichroism spectra of both folded I27-β20 (filled circles) and irreversibly unfolded ^{CM}I27-β20 (open circles) showing loss of secondary structure after carboxymethylation of the internal cysteine residues. (b) Light absorbance between 250 nm and 400 nm for 100 μM I27 (red circles), I27-β20 (green circles), ^{CM}I27 (purple circles), ^{CM}I27-β20 (blue circles).

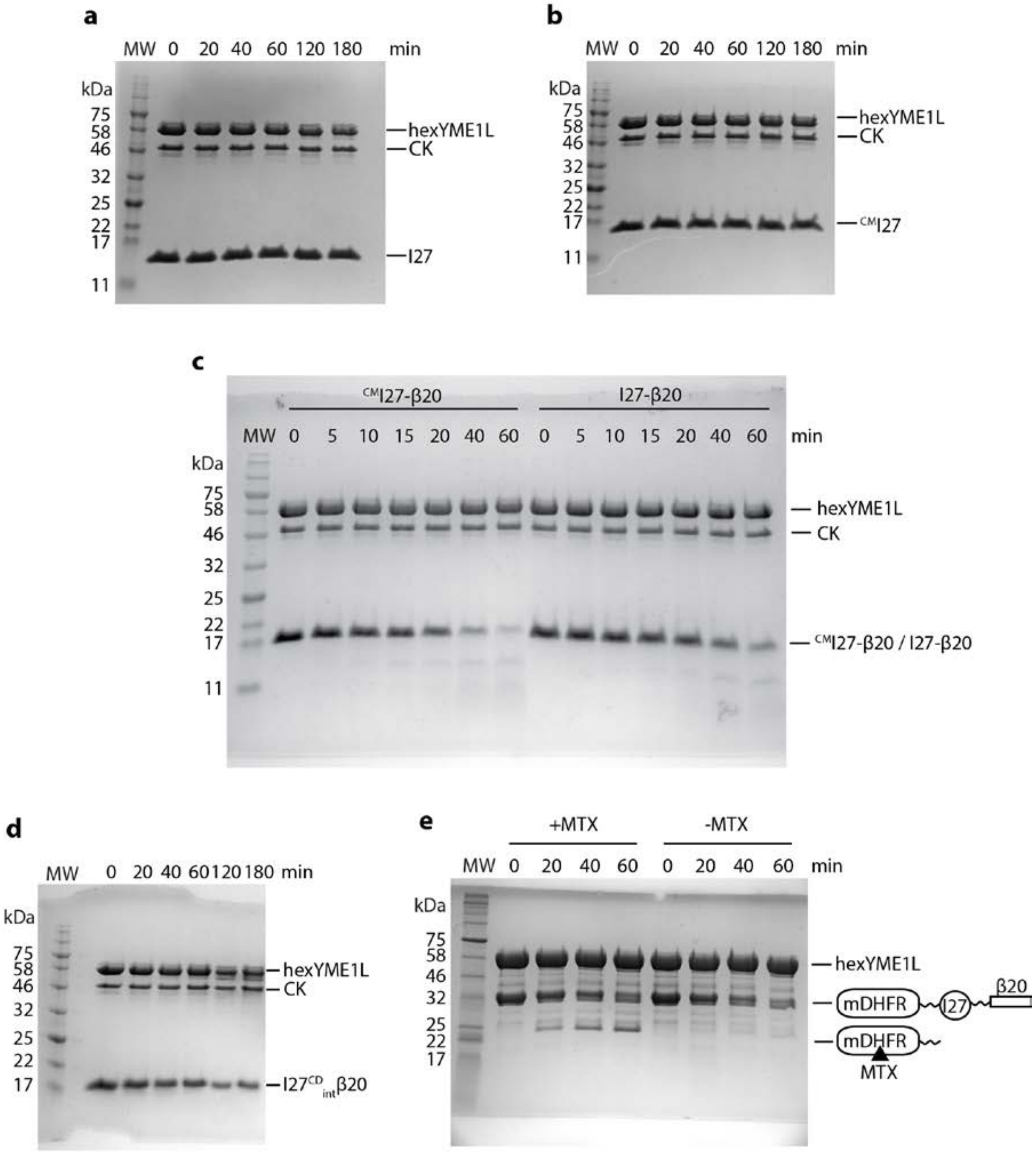

Supplementary Figure 4. Degradation of substrates in folded and unfolded states. Full SDS-PAGE images showing degradation of I27 variants from Fig. 3: (a) folded I27 lacking a degron in the presence of ATP; (b) unfolded ^{CM}I27 lacking a degron in the presence of ATP; (c) unfolded ^{CM}I27-β20 and folded I27-β20 in the presence of ATP; (d) I27$^{CD}_{int}$β20 containing an internal degron; (e) mDHFR-I27- β20 in the presence and absence of methotrexate. All degradation reactions contained 0.1 mg ml^{-1} creatine kinase (CK) as a loading control.

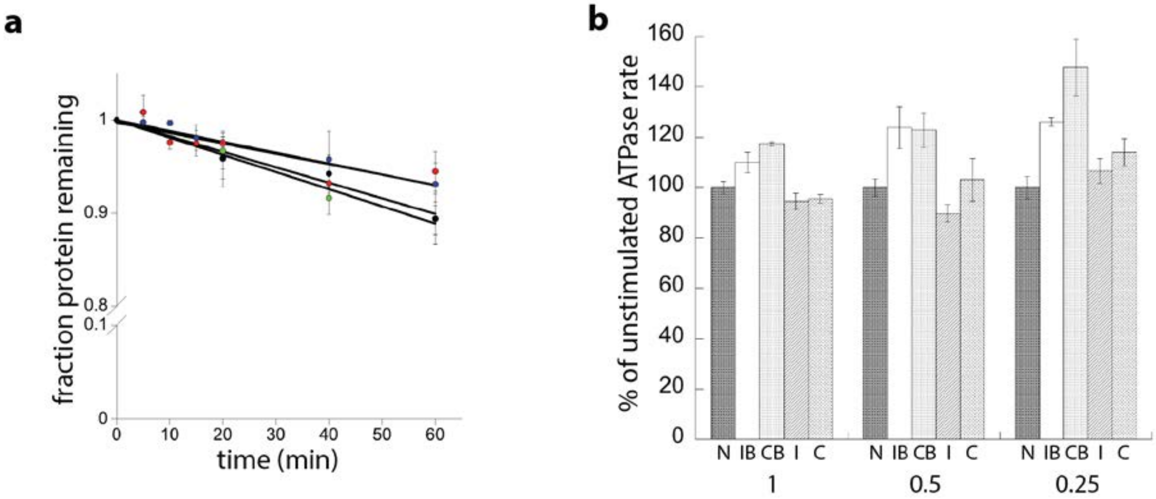

Supplementary Figure 5. Autodegradation and ATPase stimulation of hexYME1L. (a) Plot of loss of hexYME1L over time in the presence of ^{CM}I27-β20 (blue), I27-β20 (red), ^{CM}I27 (green), I27 (black). Loss of protease was quantified in the presence of ^{CM}I27-β20 or I27-β20 as less than 6 % in 1 h. (b) Plot showing relative stimulation of ATPase rate by the addition of substrates at varying enzyme concentrations. The degree of stimulation seen in the presence of no substrate (N), I27-β20 (IB), ^{CM}I27-β20 (CB), I27 (I) or ^{CM}I27 (C) alters when measured using 1 μM, 0.5 μM or 0.25 μM hexYME1L. All data shown are from independent experiments and all error bars indicate ± s.e.m (n=3).

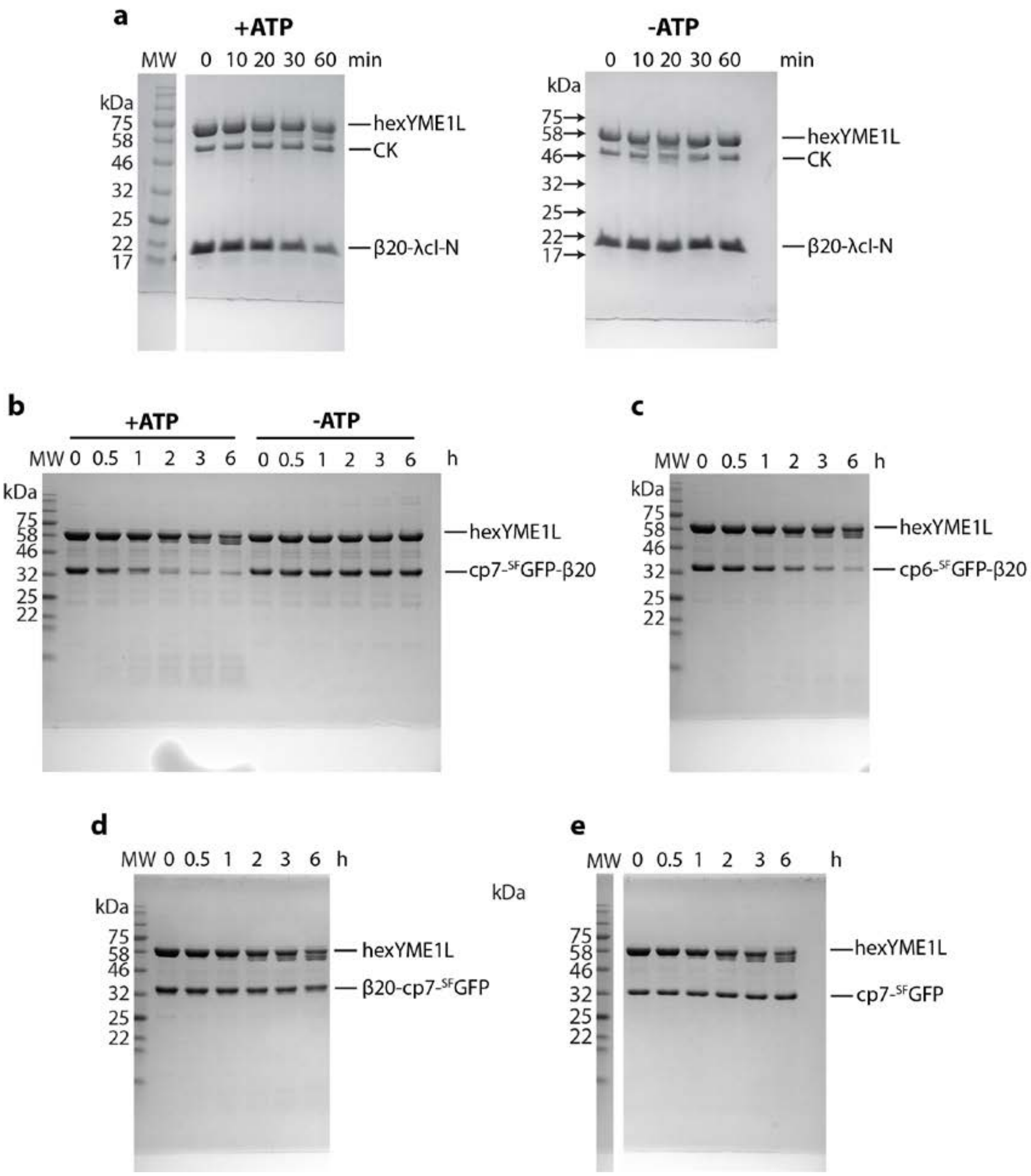

Supplementary Figure 6. Degradation of stable proteins by hexYME1L.
Full SDS-PAGE images showing degradation reactions from Fig. 4: (a) β20-λcI-N in the presence and absence of ATP; (b) cp7-^{SF}GFP-β20 in the presence of ATP; (c) cp6-^{SF}GFP-β20 in the presence of ATP; (d) β20-cp7-^{SF}GFP in the presence of ATP; (e) cp7-^{SF}GFP lacking a β20 degron in the presence of ATP.

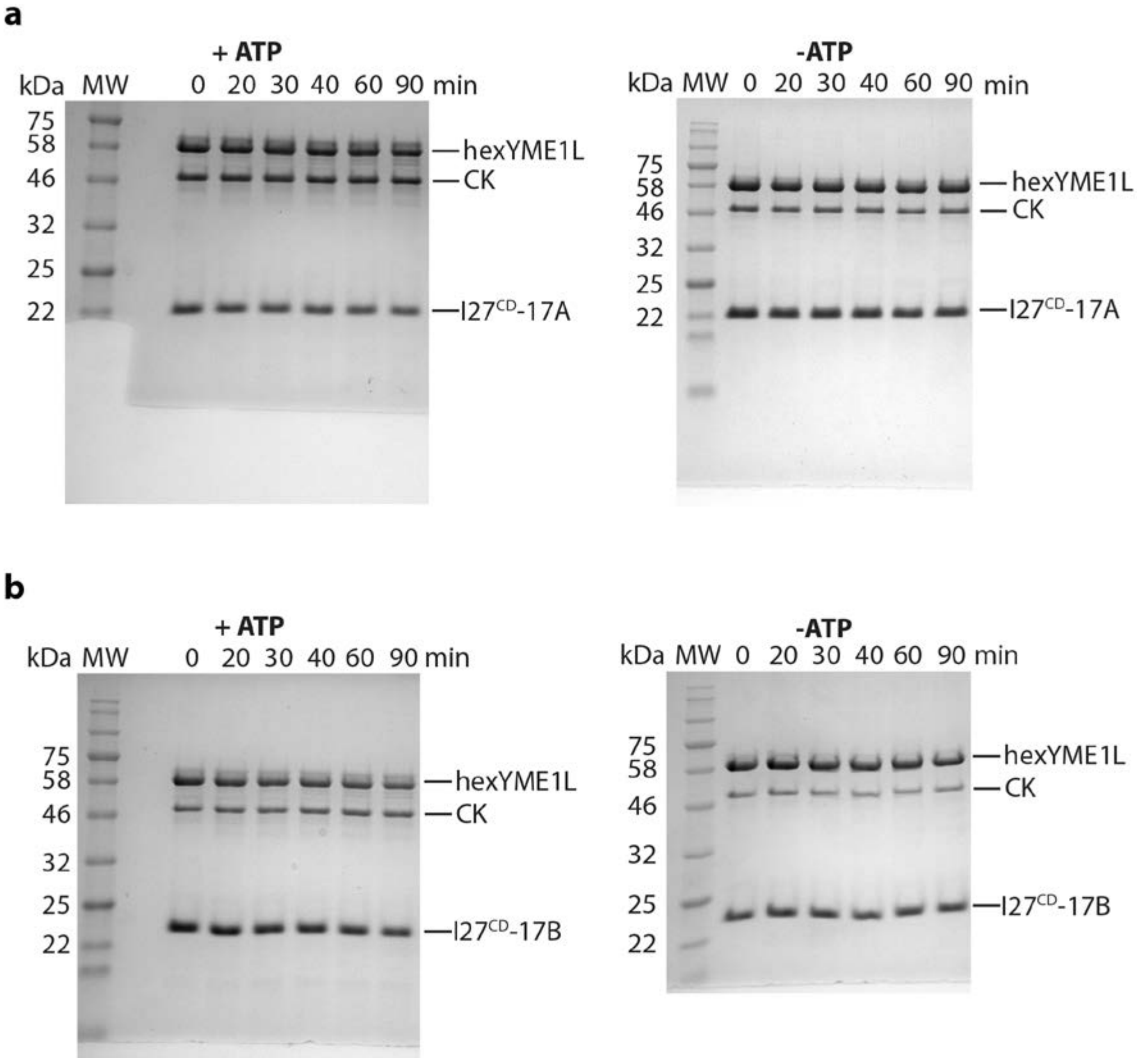

Supplementary Figure 7. Degradation of Tim17A and Tim17B by hexYME1L. Full SDS-PAGE images showing degradation of proteins containing sequences of human Tim17A and Tim17B from Fig. 5: (a) $I27^{CD}$-Tim17A in the presence of ATP (b) $I27^{CD}$-Tim17B in the presence of ATP. All reactions contained 0.1 mg ml^{-1} creatine kinase (CK) as a loading control.

Supplementary Table 1. F-h-h-F motifs identified in IMS proteins.

Uniprot ID	Name	Sequence	Location
P13073	COX41	FIGF	110-113
P36551	HEM6	FGLF	405-408
Q8TB36	GDAP1	FLGF<br>FMLF	260-263<br>337-340
Q96E52	OMA1	FVVF	206-209
O95831	AIFM1	FGGF	418-421
P00505	AATM	FAFF	239-242
Q9H078	CLPB	FLPF	568-571
Q9NRV9	HEBP1	FAVF	84-87
O43676	NDUB3	FAAF	75-78
Q6NUK1	SCMC1	FGGF	235-238
O43674	NDUB5	FGGF	29-32
O95169	NDUB8	FLAF<br>FMIF	136-139<br>139-142
P00395	COX1	FWFF<br>FLGF<br>FPLF	235-238<br>282-285<br>397-400
Q9BPX6	MICU1	FALF	417-420
P03915	NU5M	FAGF	463-466
P03905	NU4M	FYIF	118-121
O14880	MGST3	FLFF	71-74
O14949	QCR8	FVVF	53-56
Q9Y255	PRLD1	FAAF	19-22
Q14257	RCN2	FIAF	174-177
O95202	LETM1	FLVF	212-215
